# Factors associated with knowledge of hypertension among adolescents: implications for preventive education programs in primary care

**DOI:** 10.1186/s12889-015-1773-7

**Published:** 2015-05-03

**Authors:** Iga Grad, Agnieszka Mastalerz-Migas, Katarzyna Kiliś-Pstrusińska

**Affiliations:** Department of Nursing, Public Medical Academy, Opole, Poland; Department of Family Medicine, Wroclaw Medical University, Wroclaw, Poland; Department of Pediatric Nephrology, Wroclaw Medical University, Wroclaw, Poland

**Keywords:** Hypertension, Adolescents, Knowledge About Hypertension

## Abstract

**Background:**

Hypertension (HT) amongst adolescents remains a vital issue of both a medical and social nature. There is a lack of data regarding the factors influencing the awareness of the disease among the youth. The aim of this study was to evaluate the knowledge about HT among adolescents and its level corresponding to the selected demographic, environmental and medical factors.

**Methods:**

The study was carried out among 250 adolescents of secondary schools. The authors’ questionnaire poll and the psychological tests Personal Values List (PVL) and Personal Competence Scale (PCS) were performed.

**Results:**

Only 13.2% of the youth surveyed displayed the “medium” level (defined below) of HT knowledge. Most of them present satisfactory knowledge about the causes of HT. The children from urban areas generally displayed better knowledge about HT than their peers from rural regions. Only the children who had had their blood pressure previously examined displayed good knowledge about HT. The most frequently indicated source of this knowledge was school; however, its level still remains low. There was no significant association between the level of global knowledge about HT and the feeling of one’s own competences and considering the category “good health” an important personal value.

**Conclusions:**

Knowledge about HT among adolescents remains unsatisfactory and random, which indicates the necessity for routine education in this field, especially as it applies to HT symptoms. It seems that the consideration of such elements as blood pressure measurement and family history of HT in education programs can improve their efficiency.

**Electronic supplementary material:**

The online version of this article (doi:10.1186/s12889-015-1773-7) contains supplementary material, which is available to authorized users.

## Background

Hypertension (HT) is known as one of the most significant risk factors of atherosclerosis and cardiovascular diseases. HT prevalence in the worldwide adult population is estimated at 26% [1]. HT is also one of the most urgent health problems among children and adolescents [2,3]. It has been assessed that HT is common in 3.5% of the population at developmental age [4]. The highest HT frequency is observed among older children, and boys are more prone to it than girls [5]. Puberty, obesity and coexisting metabolic disorders lead to the fact that primary hypertension (PHT) becomes a dominant form of hypertension among adolescents [6]. PHT is more and more often recognized without any clinical symptoms [7]. Similarly to adults, primary hypertension among children corresponds to obesity and a family history of this disease [8-10]. Obesity, hypercholesterolemia, hypertension and habits contributing to the risk of cardiovascular diseases which have their roots in childhood, tend to continue into adulthood. Hence, any preventive actions against these diseases in adulthood will be far too late. This is why, from an early age, programs promoting physical activity, healthy eating habits and a non-smoking policy should be undertaken in order to prevent the aforementioned diseases [11-13], especially when considering the fact that more than 40% of children have complications connected with organ damage at the moment of PHT diagnosis [14]. Any preventive education program should be tailored to its recipients and their knowledge of the program’s subject. It should also correspond to the recipients’ value systems.

Taking all the evidence into consideration, we decided to carry out this study of the knowledge of HT among adolescents as well as of healthy behavior predictors in the developmental age population. The aim of our study was to obtain the answer to the following questions: a) What is the level of knowledge about HT among adolescents? b) Are selected factors such as demographic (stage of education, sex, place of living), environmental (school, family), medical (previous blood pressure measurements, a previous diagnosis of high blood pressure, family history of HT or other chronic diseases) and others related to the level of knowledge about HT among adolescents? c) Is there any relationship between the level of knowledge about HT among adolescents and factors such as the system of values or personal competence?

## Methods

The study was performed among adolescents from south-western Poland, Opole province, between April and June 2013. First, three schools were randomly selected among the 27 schools of that region (number of adolescents 11,000). Next, 300 students were randomly selected and they were invited to the study. Written informed consent was obtained from all the students. In the case of students under 18, the agreement was also signed by their parents. The study was conducted in the school during classes. Finally 250 students completed all the tests and these results were analyzed. The data concerning the demographic aspects of the tested group is presented in Table 1.

**Table 1:** **Demographic data of the examined students**

**Parameter**		**Students**
		**n = 249 (100%)**
Sex, n (%)	female	144 (57.8)
	male	105 (42.2)
Age, n (%)	16 years	32 (12.85)
	17 years	96 (38.55)
	18 years	102 (40.96)
	19 years	19 (7.63)
Secondary school grade, n (%)	I	74 (29.7)
	II	133 (53.4)
	III	42 (16.9)
Inhabited, n (%)	city (urban)	98 (39.7)
	countryside (rural)	149 (60.3)

The study was carried out on the basis of: the authors’ questionnaire and the psychological tests: Personal Competence Scale (PCS) and Personal Values List (PVL). The study protocol adhered to the Declaration of Helsinki and was approved by the Ethics Committee of Wroclaw Medical University (nr KB-378/2012).

The questionnaire consisted of questions concerning personal data, hypertension (epidemiology, causes, symptoms, complications, ways of treatment, preventive strategies), previous blood pressure measurements and HT diagnosis, as well as a family history of HT and other chronic diseases (see Additional file 1). To check if the information about HT among young people is accurate, a point scale was employed. The correct responses were given 1 point, the incorrect ones -2 points, don’t know responses or a lack of response were 0 points. The percentage ranges of were correct responses 75% and above, 74 – 50% and lower than 50%, and these were defined as good, medium and unsatisfactory knowledge, respectively. The clarity and certainty of the survey questions were confirmed in a pilot study.

### The Personal Competence Scale

The Personal Competence Scale (PCS) is designed to assess generally self-effectiveness as well as its components such as: a feeling of strength to start an action (subscale A) and prevail to continue it (subscale B). Each subscale includes 6 statements of both positive and negative meaning. The students surveyed assessed 6 of the statements applying to the fact of starting an action and 6 others applying to the frequency of given behaviors. They did it by means of choosing one out of 4 responses (respectively, *yes, rather yes, rather no,* and *no* in the former, and *almost always, often, sometimes* and *never* in the latter). Consequently, by taking them altogether, and then subscales A and B separately, the sum of the points referring to each of the responses was calculated (from 1 to 4 points, depending on how the response was formed). This made it possible to estimate a general outcome of a feeling of self-effectiveness (12–48 points) as well as the parameters of feeling strong and stubborn to complete the task (for each of 12–48 points). The raw scores of the test have been converted to sten scores 1–10 (a standard scoring system). Interpretation of the sten scores is as follows: 1–4 represents low, 5–6 medium and 7–10 high test results, respectively. To assess the feeling of strength and perseverance, higher scores go to a greater feeling of strength/perseverance and, on the other hand, lower scores refer to a lesser feeling of strength/perseverance. The PCS reliability for the total scale is 0.72, for the subscales, 0.74 and 0.62 (Cronbach’s alpha coefficient), respectively. Its theoretical accuracy ratio for total PCS is 0.43, and for subscales, 0.53 and 0.38 [15].

### The Personal Values List (PVL)

In the test, the study subjects selected 5 out of 9 symbols of happiness which they consider the most important to them. The most important symbol was given 5 points, whereas the least important one 1 point. Further on, the adolescents chose and determined the scores for 10 personal values, in accordance with the rule above. The symbols of happiness as well as personal values that were not chosen were given 0 points each. The rank assigned to “health” by the youth reflects the value which is given to “health” in the context of other individual values and possessions. The Personal Values List’s reliability is 0.78 and 0.76 for both of the parts *(test-retest).* Its theoretical accuracy is 0.74 [15].

Statistical analysis was performed with an R 2.10.1 package (for Mac OS X Cocoa GUI). Two types of Chi-square were used, compliance test schedules and test of independence. The first test verifies the null hypothesis that a frequency distribution is uniform (i.e. all entries or classes in the distribution have equal frequencies) against the alternative hypothesis that at least one is different. The test rejects the null hypothesis if the p value of significance is less than or equal to 0.05. The second test verifies the null hypothesis based on the cross tabulation (contingency tables) that the two features are independent, against the alternative hypothesis that the features are dependent. The test rejects the null hypothesis if the p value level of significance is less than or equal to 0.05. The strength of association between variables was determined by the ratio V - Cramer and Pearson's contingency coefficient. The values of both of these factors vary from 0 (no association) to 1 (complete dependence). The level of statistical significance was considered to be p-value ≤ 0.05.

## Results

### Knowledge about HT

Almost half of the adolescents questioned in this survey (49.2%) reveal a low level of global knowledge about HT. Nearly 38% of them have medium knowledge about it and only 13% have such knowledge on a good level (chi square = 50.65, df = 2, p ≤ 0.05). Knowledge of its epidemiology, ways of treatment and prevention was not satisfactory either. Most of the adolescents have good knowledge only about the causes of HT (see Table 2).

**Table 2:** **The level of partial knowledge about HT among adolescents**

**Type of knowledge about HT**	**Level of knowledge**		**Weak**	**Medium**	**Good**
			**n = 250**		
Epidemiology	Chi square = 77.86	n	144	75	31
	df = 2				
		%	57.6	30.0	12.4
	p = **0.0000**				
Causes	Chi square t = 28.38	n	65	62	123
	df = 2				
		%	26.0	24.8	49.2
	p = **0.0000**				
Symptoms	Chi square = 173.86	n	181	44	25
	df = 2				
		%	72.4	17.6	10.0
	p = **0.0000**				
Complications	Chi square = 62.05	n	94	128	28
	df = 2				
		%	37.6	51.2	11.2
	p = **0.0000**				
Ways of treatment	Chi square = 7.4	n	100	85	65
	df = 2				
		%	40.0	34.0	26.0
	p = **0.02**				
Prevention	Chi square = 12.2	n	105	85	60
	df = 2				
		%	42.0	34.0	24.0
	p = **0.002**				

HT – hypertension.

Analysis results concerning the relationships between the level of global knowledge about HT among adolescents and demographic, environmental and medical factors are presented in Table 3. Children from urban areas revealed better knowledge than their peers from rural regions (p = 0.007). Thorough analyses were devoted to the level of global and partial knowledge about HT in reference to the educational stages. Although slightly below statistical significance (p = 0.08), a relationship between the level of global HT knowledge and the level of education was found. On the other hand, a significant relationship has been shown between the level of education and the level of knowledge about HT complications (p = 0.01) and the ways of its treatment (p=0.002). It was also noted that the higher the school grade, the higher the percentage of children with better knowledge about HT.

**Table 3:** **The level of global knowledge about HT among adolescents and the chosen demographic, environmental and medical factors**

**Factor**	**Level of knowledge**		**Weak**	**Medium**	**Good**
**Sex**				n=249	
Girls	Chi square = 0.01	n	71	54	19
	df = 2				
		%	49.3	37.5	13.2
Boys		n	51	40	14
		%	48.6	38.1	13.3
	p = 1				
**Place of living**				n = 247	
Urban	Chi square = 9.77	n	36	46	16
	df = 2				
		%	36.7	46.9	16.3
Rural		n	85	47	17
		%	57.0	31.5	11.4
	p = **0.007**				
**Level of education**				n = 249	
Grade I	Chi square = 8.46	n	41	27	6
		%	55.41	36.49	8.11
Grade II	df = 4	n	65	52	16
		%	48.87	39.10	12.03
Grade III	p = 0.08	n	17	14	11
		%	40.48	33.33	26.19
**Blood pressure tests in the past**				n = 246	
No	Chi square = 14.63	n	21	4	0
	df = 2				
		%	84.0	16.0	0.0
Yes		n	98	90	33
		%	44.3	40.7	14.9
	p = **0.0006**				
**HT family history**				n = 250	
No	Chi square = 8.98	n	67	39	9
	df = 2	%	58.26	33.91	7.83
Yes		n	56	55	24
	p = **0.01**	%	41.48	40.74	17.78
**Other chronic diseases in family**				n = 250	
No	Chi square = 1.35	n	66	47	14
	df = 2				
		%	52.0	37.0	11.0
Yes		n	57	47	19
		%	46.3	38.2	15.4
	p = 0.51				
**Source of knowledge about HT**				n = 171	
School	Chi square = 20.95	n	74	68	29
	df = 2				
		%	43.3	39.8	17.0
	p = **0.0000**				
Family	Chi square = 18.77	n	46	55	18
	df = 2				
		%	38.7	46.2	15.1
	p = **0.0000**				
Internet	Chi square = 4.12	n	30	35	20
	df = 2				
		%	35.3	41.2	23.5
	p = 0.13				
Radio/TV/newspapers	C Chi square = 2.21	n	20	23	14
	df = 2				
		%	35.1	40.4	24.6
	p = 0.33				

HT – hypertension.

Our results show that there is a statistically significant positive relation between the youths’ global knowledge about HT and family history of HT (p = 0.01). The global level of knowledge of HT depending on its source is presented in Table 3. It was found that when school is taken as the main source of global HT knowledge, its level is relatively low. A similar analogy can be observed when referring to the level of knowledge about symptoms, complications and HT epidemiology. Among the people who named their families as the main source of such knowledge, there is a significant majority revealing poor global knowledge (either weak or medium). The youth also revealed a weak level of knowledge about HT epidemiology and symptoms. A good level of knowledge was found only in the causes of HT. No significant relations between the level of global knowledge about HT and the Internet (p = 0.13), the radio, newspapers or TV (p = 0.33) as a source of knowledge were found.

### Subjective factors

The analysis results regarding the level of knowledge about HT and subjective factors are presented in Tables 4 and 5. It has been shown that there was no statistically significant association between the level of global knowledge about HT, the level of knowledge about its symptoms, complications, ways of treatment and prevention and the ranges of the applied category of “good health” as the symbol of happiness. However, it was noticed that there is a tendency to display better global knowledge by the people who choose “good health” as an important symbol of happiness. Nevertheless, there is a statistically significant relationship (p = 0.05) between the level of knowledge about HT causes and the value applied to the category of “good health”. Taking all the people with good knowledge about HT causes into consideration, only 2.5% of them do not recognize “good health” as an important symbol of happiness. In the group of the people with weak and medium knowledge on this issue, the percentage mentioned above is, respectively, 14.75% and 13.11%. The precise data is presented in Table 4.

**Table 4:** **The level of global and partial knowledge about HT according to choosing “good health” as a symbol of happiness among adolescents (n = 242)**

**Level of knowledge about HT**	**Ranges**		**0**	**1**	**2**	**3**	**4**	**5**
**Epidemiological knowledge about HT**	weak	n	14	20	14	21	27	41
*Contingency coefficient C = 0.21*		%	10.22	14.60	10.22	15.33	19.71	29.93
*Cramer coefficient V = 0.15*	medium	n	5	4	6	15	19	25
*Chi square = 11.44*		%	6.76	5.41	8.11	20.27	25.68	33.78
*df = 10*	good	n	1	2	5	3	10	10
p = 0.32		%	3.23	6.45	16.13	9.68	32.26	32.26
**Knowledge about HT causes**	weak	n	9	3	4	11	16	18
*Contingency coefficient C = 0.26*		%	14.75	4.92	6.56	18.03	26.23	29.51
*Cramer coefficient V = 0.19*	medium	n	8	10	7	5	13	18
*Chi square = 18.3841*		%	13.11	16.39	11.48	8.20	21.31	29.51
*df = 10*	good	n	3	13	14	23	27	40
**p = 0.05**		%	2.50	10.83	11.67	19.17	22.50	33.33
**Knowledge about HT symptoms**	weak	n	15	23	20	28	39	51
*Contingency coefficient C = 0.19*		%	8.52	13.07	11.36	15.91	22.16	28.29
*Cramer coefficient V = 0.13*	medium	n	4	1	4	8	11	13
*Chi square = 8.94*		%	9.76	2.44	9.76	19.51	26.83	31.71
*df = 10*	good	n	1	2	1	3	6	12
p = 0.54		%	4.00	8.00	4.00	12.00	24.00	48.00
**Knowledge about HT complications**	weak	n	10	12	11	14	21	21
*Contingency coefficient C = 0.2*		%	11.24	13.48	12.36	15.73	23.60	23.60
*Cramer coefficient V = 0.14*	medium	n	10	11	12	19	26	47
*Chi square = 10.21*		%	8.00	8.80	9.60	15.20	20.80	37.60
*df = 10*	good	n	0	3	2	6	9	8
p = 0.42		%	0.00	10.71	7.14	21.43	32.14	28.57
**Knowledge about HT treatment**	weak	n	8	9	9	16	23	30
*Contingency coefficient C = 0.12*		%	8.42	9.47	9.47	16.84	24.21	31.58
*Cramer coefficient V = 0.08*	medium	n	8	10	8	14	21	22
*Chi square = 3.54*		%	9.64	12.05	9.64	16.87	25.30	26.51
*df = 10*	good	n	4	7	8	9	12	24
p = 0.96		%	6.25	10.94	12.50	14.06	18.75	37.50
**Knowledge about HT prevention**	weak	n	7	13	11	21	17	30
*Contingency coefficient C = 0.18*		%	7.07	13.13	11.11	21.21	17.17	30.30
*Cramer coefficient V = 0.13*	medium	n	9	6	9	9	23	27
*Chi square = 8.6*		%	10.84	7.23	10.84	10.84	27.71	32.53
*df = 10*	good	n	4	7	5	9	16	19
p = 0.57		%	6.67	11.67	8.33	15.00	26.67	31.67
**Global knowledge about HT**	weak	n	11	16	13	20	25	31
*Contingency coefficient C = 0.2*		%	9.48	13.79	11.21	17.24	21.55	26.72
*Cramer coefficient V = 0.14*	medium	n	9	8	10	13	20	34
*Chi square = 9.83*		%	9.57	8.51	10.64	13.83	21.28	36.17
*df = 10*	good	n	0	2	2	6	11	11
p = 0.45		%	0.00	6.25	6.25	18.75	34.38	34.38

HT – hypertension.

**Table 5:** **Results of personal competence scale among adolescents and global knowledge about HT**

**Personal competence scale**			**Global knowledge about HT**		
			**Weak**	**Medium**	**Good**
**Feeling of self-effectiveness (PWS) (n = 237)**	1-4	n	26	16	3
*Contingency coefficient C = 0.18*	low	%	57.78	35.56	6.67
*Cramer coefficient V = 0.13*	5-6	n	49	29	12
*Chi square = 8.19*	medium	%	54.44	32.22	13.33
*df = 4*	7-10	n	39	46	17
p = 0.08	high	%	38.24	45.10	16.67
**Feeling of strength (S) (n = 238)**	1-18	n	61	39	7
*Contingency coefficient C = 0.2*	lower	%	57.01	36.45	6.54
*Cramer coefficient V = 0.2*					
*Chi square = 10.09*	19-24	n	54	52	25
*df = 2*	higher	%	41.22	39.69	19.08
**p = 0.006**					
**Feeling of persistence (W) (n = 241)**	1-16	n	59	39	20
*Contingency coefficient C = 0.12*	lower	%	50.0	33.1	16.9
*Cramer coefficient V = 0.12*					
*Chi square = 3.55*	17-24	n	57	53	13
*df = 2*	higher	%	46.3	43.1	10.6
p = 0.17					

HT – hypertension.

It was observed that there is no significant relationship between the level of knowledge about HT, both on a global scale and in the aspects of its individual components, and considering the category of “good health, physical and mental efficiency” an important personal value. Similarly, it was noted that there is no significant association between the level of global knowledge about HT and a feeling of one’s own personal competences (see Table 5). It was also noted that people with a stronger feeling of strength possess vividly statistically better knowledge than the ones with a weaker feeling of strength (p = 0.006). Such a relationship was not observed when referring to a feeling of persistence. It was noticed as well that the percentage of people with a higher feeling of strength and good knowledge about HT treatment is much higher (31.6%) than the percentage of the youth with a lower feeling of strength and good level of knowledge (p = 0.05).

## Discussion

The results of our study have shown that the youths’ knowledge about HT is both unsatisfactory and random, which indicates the necessity of its promotion across this age group, especially in the field of its symptoms. According to the research performed by various authors, general knowledge among young people, when applied to noninfectious diseases, is very poor [16,17]. Among others, Vanhecke et al. [18] demonstrated that adolescents lack knowledge regarding the risk of cardiovascular disease and this consequently corresponds with our own observations as far as the issue of HT is concerned.

The research has not revealed any significant relationship between the youths’ knowledge about HT and their age. However, the higher the level of education, the higher the percentage is of adolescents presenting good knowledge about this disease, whereas the percentage of young people with poor knowledge about it decreases. Such a tendency is in accordance with a commonly-known concept claiming that children’s knowledge expands along with increasing years of education [19-21].

The results of our own research do not show any differences in global knowledge about HT when taking sex into consideration. As for other authors, their observations in this field are varied [18,22,23]. Our research has also shown that urban adolescents have better knowledge about HT than their peers from rural areas. The explanation of these results lies in the diversification of learning progress, which is varied for children of different regions and different social backgrounds [24]. Adolescents of rural areas tend to obtain lower rates in an intellectual learning process. Therefore, during educational activities, it is worth paying attention to the fact that the acquisition of knowledge about health and diseases might become more difficult for such groups of students.

School is reported to be perceived by the youth as the main source of knowledge about HT. This is why such a low level of global knowledge about this issue is alarming, as it indicates certain teaching shortcomings in educational institutions. Our research has also revealed the fact that a family is not thought to be a source of a good knowledge about HT either. Such an unsatisfactory level of knowledge indicates that, despite their sources, all the health messages concerning HT show visible weaknesses. It might be connected with their content, form and ways of transmission or ultimately their inadequate adaptation to a specific group of receivers. All of the factors shown above indicate the need for modification of both the content and forms of educational undertakings.

The current study has revealed as well that the students who previously had had blood pressure measurements possess twice the degree of knowledge about HT than their peers who have not had such tests so far. In fact, none of this group is reported to have a good a level of knowledge about it. The results imply the necessity of conducting blood pressure tests during check-ups in medical centers and putting practice and theory on HT into one.

It is known that an individual’s knowledge is best put into practice only if the subject of knowledge applies to the system of a given person’s value. The results of our study show that 20% of young people fail to consider health to be a component of their system of important personal values. On the other hand, almost one in three of the surveyed youth admitted that health is the most important symbol of happiness. According to our own analysis as well as the opinions given by healthcare experts, young people do not consider health a value itself but treat it rather as a way to obtain happiness in their lives. Such an approach may lead to undertaking activities which might cause harm to their health [24]. The perception that health is merely a product to be purchased brings passiveness and a lack of remorse and motivation to actions whose aim is to both maintain and strengthen their health condition. It also causes a lack of responsibility for their health and lifestyles. This is why any educational activities should include making adolescents more aware of the value of health in their lives. Such consciousness may influence their feelings of the realization of their health and treating their health self-care in terms of a necessity, not only an obligation.

The starting of a specific activity by a person depends on one’s beliefs about one’s ability to influence the course of action. In our study, four out of ten of those surveyed reported a feeling of high self-effectiveness, and nearly two in ten revealed low competence markers. In spite of the fact that no significant association was observed between a feeling of self-effectiveness among students and their knowledge about HT, we can see a tendency among the youth to have better knowledge about HT only if it is accompanied by an increase in self-effectiveness. The relatively high self-efficacy in the examined group of adolescents can be considered a positive factor in the prevention of youth health issues. It has a positive effect on the processing of information, formulating goals, undertaking tasks and their execution [25,26]. A sense of one’s own self-efficacy will therefore be important for the implementation of preventive educational programs.

The conducted study has some limitations. First, the effective sample size of the study, although selected from among a large number of secondary school students, is small, implying a limited statistical power. Our sample was derived from one region of Poland, therefore the observations captured herein can only be generalized to settings with a similar context. However, it is likely that similar processes exist in other parts of Poland and the results of this study could be used as a basis for a larger survey which could attempt to confirm them in a broader setting.

In our study, we analyzed, among others, the association between the level of knowledge about HT among adolescents and their system of values, including health. Taking into account different systems of values could show other results. Although all of the students completed the survey unassisted in their schools, we can’t exclude the impact of location and context on choices made. Finally, we did not analyze many other factors that may be related to the knowledge of HT and may be useful in designing preventive strategies in an adolescent population, so further studies are needed in this area.

## Conclusions

Knowledge about HT among adolescents remains unsatisfactory and random, which indicates the necessity for routine education in this field, especially as it applies to HT symptoms. It seems that the consideration such elements as blood pressure measurement and family history of HT in education programs can improve their efficiency. Particular attention should be paid to the strengthening of "health" as a personal value and to build young people's sense of personal competence for the creation and implementation of healthy behavior.

## Additional file

Additional file 1:
**The authors’ questionnaire.**

## Abbreviation

HT: Hypertension
PHT: Primary hypertension
BP: Blood pressure
