# Supplementary material for: Factors associated with knowledge of hypertension among adolescents: implications for preventive education programs in primary care
Source: BMC Public Health. 2015 May 3;15:463. doi: 10.1186/s12889-015-1773-7 (PMC4422228; doi:10.1186/s12889-015-1773-7)
Supplement: Additional file 1: — The authors’ questionnaire. [file 12889_2015_1773_MOESM1_ESM.doc]

**QUESTIONNAIRE**

| **A. Initials** | ………………………………………….. |
| --- | --- |
| **B. Sex** | 1. female  2. male |
| **C. Age**  *(Please enter your age in years)* | ………………………. |
| **D. Secondary school grade** | I  II  III |
| **E. Place of residence** | 1. city  2. village |

**Please select one or more answers.**

**Please fill in the questionnaire honestly.**

| **1. Do you know the definitions: blood pressure, hypertension?** | 1. yes  2. no |
| --- | --- |
| **2. Do children and adolescents suffer from hypertension?** | 1. yes  2. no, it occurs only among adults  3. do not know |
| **3. If children and adolescents suffer from hypertension, what is the age of disease manifestation:** | 1. 0-3 years 2. 4-8 years 3. 9-12 years 4. 13-16 years 5. more than 16 years 6. all ages 7. do not know |
| **4. If children and adolescents suffer from hypertension, in which group does it occur predominantly?** | 1. 0-3 years 2. 4-8 years 3. 9-12 years 4. 13 years and more 5. all age ranges with similar frequency 6. do not know |
| **5. Which factors can lead to hypertension?**  *(please mark “X” )*   |  | YES | NO | DO NOT KNOW | | --- | --- | --- | --- | | a) genetic factors |  |  |  | | b) unhealthy diet – crisps, fast-food, a salt surfeit |  |  |  | | c) overweight and obesity |  |  |  | | d) low physical activity |  |  |  | | |
| **6. Do the following conditions lead to hypertension?**  *(please mark “X”)*   |  | YES | NO | DO NOT KNOW | | --- | --- | --- | --- | | a) tobacco smoking |  |  |  | | b) alcohol abuse |  |  |  | | c) drugs, doping substances, substances contained in energizing products |  |  |  | | d) rivalry tendency, hostility, impatience, haste |  |  |  | | |
| **7. Correct blood pressure values for adults are:** | 1. under 120/80 mmHg  2. under 140/90 mmHg  3. 160/90 mmHg  4. others, please specify ........................................  5. do not know |
| **8. What are the symptoms of hypertension among children/adolescents?** | 1. headache 2. palpitations 3. easy fatigue 4. visual disturbances   5. others, please specify ..........................................................   1. hypertension may not have any symptoms 2. do not know |
| **9. Except hypertension as a disease itself, can elevated blood pressure be a symptom of other illness?** | 1. yes, e.g. kidney, heart, thyroid disease 2. no 3. do not know |
| **10. The correct blood pressure values for children and adolescents are:** | 1. the same as in adults 2. various, depending on the age 3. do not know |
| **11. Can hypertension cause complications - various organ damages?** | 1. yes 2. no 3. do not know |
| **12. If you have chosen „1” in point 11, please select diseases/disturbances caused by hypertension:** | 1. heart diseases 2. cardiovascular diseases 3. stroke 4. kidney diseases 5. visual disturbances 6. others, please specify ................................................. 7. do not know |
| **13. What does a hypertension treatment involve?** | 1. pharmacotherapy 2. body weight standardization 3. controlled physical activity 4. lifestyle changes 5. others, please specify........................................................ 6. do not know |
| **14. Lifestyle changes include:**  *(please mark “X”)*   |  | YES | NO | DO NOT KNOW | | --- | --- | --- | --- | | diet modification: reducing salt intake, reducing intake of fatty foods |  |  |  | | stop tobacco smoking |  |  |  | | not to use drugs, doping substances, energizing products |  |  |  | | psychotherapy |  |  |  | | |
| **15. Can hypertension development be stopped?** | 1. yes, always 2. in some cases 3. no 4. do not know |

| **16. If you have chosen 1 or 2 in question above, please mark ways of hypertension prophylaxis:** | 1. proper nutrition 2. body weight maintenance 3. regular physical activity 4. not to tobacco smoking 5. stopping of alcohol abuse 6. elimination of hurry, rivalry, enmity response, impatience 7. others, please specify ................................................... 8. do not know |
| --- | --- |
| **17.** **What is the source of your knowledge about hypertension?** | 1. school 2. family 3. friends 4. internet 5. radio/TV/newspapers 6. other, please specify ................................................ |
| **18. Have you ever had blood pressure measured?** | 1. yes 2. no |
| **19. If you marked “yes” in question above, please specify:**  A. when *(year of life)*:  B. where: | ……………………………………………..   1. outpatient department 2. hospital 3. school nurse office 4. at home 5. other, please specify .............................. |
| **20. Have you ever had elevated blood pressure?** | 1. yes 2. no |
| **21. If you marked “yes” in question above please specify:**  A. when *(year of life)*:    B. where: | ………………………………………………….   1. outpatient department 2. hospital 3. school nurse office 4. at home 5. other place, please specify.................................. |
| **22. Do you suffer from hypertension?** | 1. yes 2. no |
| **23. If you have hypertension please specify:**  A. time of diagnosis  *(year of life)*:  B. where diagnosis was obtained:  C. what kind of treatment is used: | …………………………………………..   1. outpatient department 2. hospital 3. other place, please specify......................................   ……………………………………………………………………………………………………………… |
| **24. Do your relatives suffer from hypertension?** **If yes, please indicate:** | 1. parents 2. siblings 3. grandparents 4. cousins 5. do not know 6. nobody suffers from hypertension |
| **25. Do your relatives suffer from chronic diseases? If yes, please indicate:** | 1. heart diseases 2. diabetes 3. musculoskeletal diseases 4. obesity 5. kidney diseases 6. others, specify what kind ……………………… 7. do not know 8. nobody suffers from chronic diseases |

***Thank you for your time and attention.***
